# Epidemiological Profile and Outcome of COVID-19 Patients and Its Comparison Between the First and Second Wave at a Tertiary-Level COVID Care Facility in North India

**DOI:** 10.7759/cureus.74456

**Published:** 2024-11-25

**Authors:** Prabhaker Mishra, Ratender K Singh, Alok Nath, Om Prakash Sanjeev, Zia Hashim, Radha K Dhiman, Shantanu Pande, Tanmoy Ghatak, Amit Rastogi, Devendra Gupta

**Affiliations:** 1 Biostatistics and Health Informatics, Sanjay Gandhi Post Graduate Institute of Medical Sciences (SGPGIMS), Lucknow, IND; 2 Emergency Medicine, Sanjay Gandhi Post Graduate Institute of Medical Sciences (SGPGIMS), Lucknow, IND; 3 Pulmonary Medicine, Sanjay Gandhi Postgraduate Institute of Medical Sciences (SGPGIMS), Lucknow, IND; 4 Emergency Medicine, Sanjay Gandhi Postgraduate Institute of Medical Sciences (SGPGIMS), Lucknow, IND; 5 Hepatology, Sanjay Gandhi Postgraduate Institute of Medical Sciences (SGPGIMS), Lucknow, IND; 6 Cardiovascular Surgery, Sanjay Gandhi Post Graduate Institute of Medical Sciences (SGPGIMS), Lucknow, IND; 7 Anaesthesiology, Sanjay Gandhi Post Graduate Institute of Medical Sciences (SGPGIMS), Lucknow, IND

**Keywords:** clinical-demographic, comorbidity, covid-19, first and second wave, mortality, respiratory support

## Abstract

Background: In December 2019, COVID-19 emerged in China and spread rapidly throughout the world, including India. So far, India has witnessed three spells of the disease, termed the first, second, and third waves; although the first two waves were significant in terms of severity, mortality, and need for respiratory support, the third wave had no significant impact and most people recovered without being admitted to the hospital. The present study aimed to discuss the clinical demographic characteristics and in-hospital outcomes of COVID-19 patients and their comparisons between the first and second waves.

Methods: This was a retrospective, single-center study, comprising data collected regarding demographic, epidemiological, and outcomes of COVID-19 patients admitted to a tertiary care facility in north India. Patient death or requirement of respiratory support was considered as patient outcomes.

Results: Of 3563 COVID-19 patients, 2459 comprised the first wave and 1104, the second wave. There was a significantly higher age, increased proportion of female gender, clinical symptoms, ventilator, and non-invasive ventilation (NIV) support, as well as patients’ mortality in the second wave, whereas the distribution of the comorbidities was statistically equal between the first and second waves.

Conclusions: The second wave of COVID-19 was more devastating in terms of the severity of the disease, the need for respiratory support, and mortality in the patients. Poor outcomes were associated with the presence of any one comorbidity and the absence of vaccination in the patients.

## Introduction

On December 31, 2019, the World Health Organization (WHO) informed the world about cases of pneumonia of unknown cause in Wuhan City, Hubei Province, China. On January 7, 2020, researchers identified a novel coronavirus and temporarily named it "2019-nCoV" [1]. This virus spread speedily worldwide, and the WHO subsequently declared coronavirus disease 2019 (COVID-19) a global pandemic on March 11, 2020 [2]. We detected more than 21.4 crore COVID-19 cases worldwide until August 27, 2021, with 44.7 lakh deaths (2.1% of total cases). During the period, India detected 3.26 crore cases, with a case fatality rate of 1.34% [3].

On January 27, 2020, in Kerala, a 20-year-old female presented with a history of dry cough and sore throat, marking the first case of COVID-19 in India [4]. Following the index case, there was a gradual surge of cases between March 2020 and September 2020, with the highest number of new cases detected on 16th September 2021 (n=97894). Following this, the number of cases exhibited a downward trajectory, reaching a nadir on February 8, 2021 (n=9110). However, a second upsurge was witnessed in March 2021, culminating in a peak of 4,14,188 new cases recorded on May 6, 2021. The trends showed a downward slope after this second peak with cases, with new cases ranging between 30 and 50 thousand per day till the end of August 2021 [5].

The state of Uttar Pradesh, having the largest populace in India, witnessed fluctuations in COVID-19 cases proportionate to the national trends. It reported its first case in March 2020 and witnessed two peaks in September 2020 and April 2021, respectively. From March 2020 to February 2021, cases were mainly due to the SARS-CoV-2 strain, and after March 2021 onwards, it was due to a new variant of the same virus [6]. On account of the variant strain, with higher reported infectivity in Uttar Pradesh, in consonance with the national trajectory, witnessed an unprecedented upsurge in hospitalization and proportion of severe COVID-19 cases requiring respiratory support. Based on the time-based trajectory of cases and the emergence of variant strains, the phase between March 1, 2020 and February 28, 2021 has been considered the first COVID-19 wave. In the Indian context, we classify the period from March 1, 2021 to June 30, 2021 as the second wave. Literature regarding the differences in demography, symptomatology, and outcomes between the two waves is limited. Given the dynamicity of COVID-19, it is important to explore the differential aspects between the two waves, which prompted us to compare the demographic and epidemiological characteristics of the COVID-19 cases between the first and second waves and their association with patient outcomes in terms of respiratory support requirement and patients’ mortality.

## Materials and methods

Study setting and design

This was a hospital-based analytical observational study based on retrospective data from patients treated at Rajdhani COVID-19 Hospital, a dedicated level III COVID-19 care facility of the Sanjay Gandhi Postgraduate Institute of Medical Sciences, Lucknow, India. All consecutive COVID-19 patients (N=3563) confirmed by RT-PCR and discharged after treatment or having an adverse outcome between March 21, 2020 and June 30, 2021 were enrolled for the study. The study was performed after approval from the institute ethics committee (2021-232-IP-EXP-42).

Data sources and measurements

Patient data (stratified as non-survivors [NSs] and survivors [Ss]) was retrospectively collected and extracted from the discharge summaries and hospital information system (HIS), which is an electronic database of patients available at the institute of intranet [7]. The extracted data were entered and reviewed by a team of clinicians and biostatisticians. In case the same patient was admitted more than once, only the latest admission was considered for the study. Demographic data of the patients, including age (years) at admission, sex and clinical symptoms (fever, dry cough, sore throat, breathlessness, or shortness of breath), were recorded for the study. Symptoms were recorded as per the Center for Disease Control and Prevention definitions [6]. Associated co-morbidities like diabetes, hypertension (HTN), renal, heart, lung, liver, malignancy, and any other significant co-morbidities were also recorded. The criteria for discharge were at least two RT-PCR negative test results, and in cases of differing test results, a third RT-PCR negative result was required.

Variables

We compared the demographic, epidemiological profile, and patient outcomes between the two waves. The primary outcome was defined as clinical recovery from COVID-19 at the time of hospital discharge or death, whereas secondary objectives were required respiratory support.

COVID-19 scoring system

Based on demographic and epidemiological variables, a risk score was developed named “COVID-19 Scoring System (CSS)” [8]. In this scoring system, based on five identified independent predictors (age ≥60 years: score 2, breathlessness: score 3, cough: score 2, diabetes mellitus: score 1 and any other comorbidities: score 1), a score was developed. The developed risk score ranged from 0 to 9, where a higher score indicates a higher risk for mortality. Further risk scores were divided into three groups: 0-4 (mild), 5-7 (moderate) and 8-9 (severe) [8]. In the present study, this CSS was used to divide patients into low, medium, and high-risk groups.

Sample size

At minimum two-sided 95% confidence interval and 90% power of the study, assuming a 6% (15% vs. 21%) difference in mortality (estimated sample size was 1599 and 799 in the first and second wave, respectively) and 6% difference in need of respiratory support (50% vs. 56%) (estimated sample size was 2177 and 1088 in the first and second wave, respectively). Finally, 2459 and 1104 patients were taken in the first and second waves, respectively. The sample size was estimated using the software Power Analysis and sample size version 16 (PASS-16, NCSS, LLC, Utah, USA).

Statistical analysis

Continuous variables are presented in the median (interquartile range, "IQR"), whereas categorical variables are in number (%). The Mann-Whitney U test was used to compare the medians, whereas the chi-square test was used to compare the proportion between the first and second waves. Univariable or multivariable binary logistic regression analysis was used to test the association between mortality or need for respiratory support with the CSS and vaccination (in the second wave only) for the first and second waves. Classification and regression trees (CART) analysis was used to present the distribution of the patient mortality or need for respiratory support as per the CSS and vaccination. A P-value <0.05 was considered statistically significant. Statistical package for social sciences, version 23 (SPSS-23, IBM, Chicago, USA) and MedCalc Statistical Software, version 20 (Ostend, Belgium) were used for data analysis.

## Results

A total of 3563 COVID-19 patients (67.6% males, n=2408) were included, with a mean and median age of 51.87 and 54 years, respectively. 47.5% (n=1693) required respiratory support during treatment. The overall mortality (non-survivors) was 18.5% (n=659). 

Of the 3563 patients, 2459 were admitted during the first wave and 1104 in the second wave. The proportion of females increased in the second wave (31.4% vs. 35%, p<0.05). Patients in the second wave were older (median: 53 vs. 55 years, p<0.05). The cumulative requirement of invasive and non-invasive ventilation (NIV) was higher in the second wave (16.06% vs. 26.54%, p<0.001). However, a requirement of only oxygen support (without invasive or non-invasive ventilation) (32.4% vs. 28%, p<0.05) as well as non-requirement of any respiratory support (51.6% vs. 45.5%, p>0.05) was lower in the second wave. No vaccinated patients were admitted to this facility during the first wave (concordant with the onset of vaccination availability). Of all the patients admitted in the second wave, 37.8% were vaccinated.

When stratified as per symptomatology, a higher proportion of patients reported clinical symptoms in the second wave [fever (64.1% vs. 80.6%, p<0.001), breathlessness (33.2% vs. 42.3%, p<0.001), cough (39.2% vs. 45.5%, p<0.001), and sore throat (16.7% vs. 24.5%, p<0.001)]. 

The presence of hypertension and diabetes were almost similar between both waves. Co-morbidities in the form of renal (15% vs 11.7%, p<0.05), cardiac (8.5% vs 5.9%, p<0.05), lung (10% vs 8%, p<0.05), liver (2.5% vs 0.5%, p<0.05), and cancer (2.6% vs 1.0%, p<0.05) were higher in the first wave. Contrastingly, patients with other comorbidities (including asthma, obesity, etc.) were higher (6.5% vs. 15.2%, p<0.05) in the second wave. When stratified as per the number of co-morbidities (one, two, or three), patients in both waves were similar. When co-morbidities were combined, a combination of diabetes, hypertension, and heart disease (4.3% vs. 2.8%, p<0.05) and a combination of diabetes, hypertension, and renal disease (4.6% vs. 2.9%, p<0.05) was higher in the first wave.

Duration of hospital stay was significantly lower (median days: 12 vs. 11, p<0.001) in the second wave compared to the first wave. Table 1 displays the patient characteristics.

**Table 1:** Distribution of the demographic, respiratory support requirement, clinical symptoms, comorbidities, and mortality between first and second waves (N=3563) DM: diabetes mellitus; HTN: hypertension; Renal: kidney disease; Heart: heart disease. Age and hospital stay were presented in the ^#^median (first quartile, third quartile) compared by the Mann-Whitney U test. All other variables are presented in numbers (column %) compared by the chi-square test. Z and χ2 test statistics were calculated for the Mann-Whitney U test and chi-square test, respectively. P<0.05 significant.

Variables	Total (N=3563)	1^st^ wave (n_1_=2459)	2^nd^ wave (n_2_=1104)	Z or χ^2^ value	P-value
Demographic variables					
Age (years)^#^	54(40.63)	53(40.63)	55(43.63)	3.09	0.002
Age groups				17.31	<0.001
<18 years	76(2.2)	69(2.8)	7(0.6)	17.78	<0.001
18-60 Years	2282(64)	1568(63.8)	714(64.7)	0.27	0.605
≥60 years	1205(33.8)	822(33.4)	383(34.7)	0.58	0.448
Female sex	1155(32.4)	764(31.1)	391(35.4)	6.42	0.011
Hospital stay days^#^	12(8.17)	12(9.17)	11(7.16)	6.53	<0.001
Respiratory support					
Invasive ventilator	540(15.2)	306(12.4)	234(21.2)	45.97	<0.001
Non-invasive ventilator	148(4.2)	89(3.6)	59(5.3)	5.57	0.018
Oxygen	1105(31)	796(32.4)	309 (28)	6.92	0.009
No support	1770(49.7)	1268(51.6)	502(45.5)	11.36	<0.001
Vaccination					
None	687(62.2)	0	687(62.2)	-	-
Partial	328(29.7)	0	328(29.7)	-	-
Complete	89(8.1)	0	89(8.1)	-	-
Clinical symptoms					
Fever	2467(69.2)	1577(64.1)	890(80.6)	97.42	<0.001
Breathlessness	1284(36)	817(33.2)	467(42.3)	27.35	<0.001
Cough	1467(41.2)	965(39.2)	502(45.5)	12.46	<0.001
Sore throat	680(19.1)	410(16.7)	270(24.5)	30.03	<0.001
Comorbidity					
HTN	1352(37.9)	935(38.1)	417(37.8)	0.03	0.865
DM	1229(34.5)	862(35.1)	367(33.2)	1.23	0.270
Renal disease	499(14.0)	370(15.0)	129(11.7)	6.90	0.009
Heart disease	275(7.7)	210(8.5)	65(5.9)	7.24	0.007
Lung disease	335(9.4)	247(10.0)	88(8.0)	3.57	0.058
Liver disease	66(1.9)	61(2.5)	5(0.5)	15.05	<0.001
Cancer	75(2.1)	64(2.6)	11(1.0)	9.49	0.002
Others	329(9.2)	161(6.5)	168(15.2)	69.06	<0.001
DM + HTN	783(22)	558(22.7)	225(20.4)	2.34	0.125
DM + HTN + renal	144(4)	112(4.6)	32(2.9)	5.62	0.018
DM + HTN + heart	136(3.8)	105(4.3)	31(2.8)	4.67	0.031
DM + HTN + heart + renal	25(0.7)	17(0.7)	8(0.7)	0	0.99
At least one comorbidity	2281(64)	1596(64.9)	685(62)	2.79	0.095
No. of comorbidity					
None	1282(36)	863(35.1)	419(38)	2.79	0.095
One	1005(28.2)	706(28.7)	299(27.1)	0.96	0 .326
Two	787(22.1)	544(22.1)	243(22)	0.00	0.947
Three	383(10.7)	270(11)	113(10.2)	0.50	0.476
≥Four	106(3.0)	76(3.1)	30(2.7)	0.42	0.516
Outcome (deaths)	659(18.5%)	377(15.3)	282(25.5)	52.71	<0.001

Comparison of need for respiratory support and mortality between first and second waves

We compared the need for respiratory support and mortality between the two waves. Both respiratory support requirements (48.4% vs. 54.5%, p<0.001) and mortality (15.3% vs. 25.5%, p<0.001) were significantly lower in the first wave. Patients with at least one comorbidity had higher respiratory support requirements (53.3% vs. 61.2%, p<0.001) and mortality (21.4% vs. 31.1%, p<0.001) in the second wave. There were no vaccinated patients in the first wave, whereas in the second wave, 417 of 1104 (37.8%) patients (29.7% single dose and 8.1% double dose) were vaccinated. The requirement for respiratory support (44.8% vs. 57.1%) and mortality (15.3% vs. 31.7%) in vaccinated patients was lower than in non-vaccinated patients. There was a higher percentage of patients who reported respiratory support and deaths within 24 hours during the second wave (Tables 2-3).

**Table 2:** Association of various factors with respiratory support used and its comparison between first and second wave (N=3563). DM: diabetes mellitus, HTN: hypertension. Renal: kidney disease, Heart: heart disease. Categorical variables presented in number (row%) and respiratory support were compared between two waves by chi-square test. χ2 test statistics were calculated for the chi-square test. P<0.05 significant.

Variables	Respiratory Support Required					
	1st Wave		2nd Wave		χ2 value	P value
	Total Patients	Respiratory Support: number (%)	Total Patients	Respiratory Support: number (%)		
Patients	2459	1191(48.4)	1104	602(54.5)	11.36	<0.001
Age groups						
<18 Years	69	24(34.8)	7	2(28.6)	0.11	0.743
18-60 Years	1568	682(43.5)	714	358(50.1)	8.61	0.003
≥60 Years	822	485(59)	383	242(63.2)	1.92	0.165
Sex						
Male	1695	847(50)	713	391(54.8)	4.62	0.008
Female	764	344(45)	391	211(54)	8.39	<0.001
Vaccinated #						
Yes	0	0	417	187(44.8)	--	-
No	2459	1190(48.4)	687	415(60.0)	24.11	<0.001
Comorbidity						
Diabetes DM)	862	510(59.2)	367	234(63.8)	2.28	0.131
Hypertension (HPT)	935	514(55)	417	255(61.2)	4.52	0.034
Renal	370	186(50.3)	129	83(64.3)	7.53	0.006
Heart	210	133(63.3)	65	51(78.5)	5.16	0.023
Lung	247	172(69.6)	88	72(81.8)	4.86	0.028
Liver	61	39(63.9)	5	4(80)	0.519	0.471
Cancer	64	21(32.8)	11	5(45.5)	0.66	0.416
Other Comorbidities	161	64(39.8)	168	104(61.9)	16.02	<0.001
DM+HTN	558	335(60)	225	142(63.1)	0.646	0.422
DM+HTN+ Renal	112	72(64.3)	32	25(78.1)	2.14	0.143
DM+HTN+Heart	105	70(66.7)	31	20(64.5)	0.051	0.821
DM+HTN+Heart+ Renal	17	9(52.9)	8	6(75)	1.06	0.303
At least one comorbidity	1596	850(53.3)	685	419(61.2)	12.11	<0.001
Number of Comorbidities						
Nil	863	341(39.5)	419	183(43.7)	2.06	0.152
One	706	332(47)	299	167(55.9)	6.65	0.009
Two	544	298(54.8)	243	146(60.1)	1.92	0.165
Three	270	171(63.3)	113	80(70.8)	1.98	0.160
Four	76	49(64.5)	30	26(86.7)	5.08	0.024
Duration of Hospital stay						
24 hours	21	9(42.9)	13	10(76.9)	3.65	0.056
24-72 hours	105	58(55.2)	80	48(60)	0.425	0514
4-7 days	302	144(47.7)	230	99(43)	1.16	0.281
≥7 days	2031	980(48.3)	781	445(57)	17.08	<0.001

**Table 3:** Association of various factors with mortality and its comparisons between first and second wave (N=3563). DM: diabetes mellitus; HTN: hypertension; NIV: non-invasive ventilation. Renal: kidney disease, Heart: heart disease. Categorical variables are presented in number (row %) and mortality was compared between two waves by Chi-square test. χ2 test statistics were calculated for the chi-square test. P<0.05 significant.

Variables	Mortality					
	1st wave		2nd wave		χ2 value	P-value
	Total Patients	Mortality: number (%)	Total patients	Mortality: number (%)		
Patients	2459	377(15.3)	1104	282(25.5)	52.7	<0.001
Age groups						
<18 Years	69	6(8.7)	7	1(14.3)	0.25	0.63
18–60 Years	1568	149(9.5)	714	144(20.2)	50.2	<0.001
≥60 Years	822	222(27)	383	137(35.8)	9.66	0.001
Sex						
Male	1695	285(16.8)	713	194(27.2)	34.01	<0.001
Female	764	92(12)	391	88(22.5)	21.69	<0.001
Respiratory support						
Ventilator	306	292(95.4)	234	224(95.7)	0.29	0.87
NIV	89	58(65.2)	59	28(47.5)	4.54	0.033
Oxygen	796	12(1.5)	309	25(8.1)	29.50	<0.001
No Support	1268	15(1.2)	502	5(1.0)	0.13	0.723
Vaccinated#						
Yes	0	0	417	64(15.3)	-	-
No	2459	377(15.3)	687	218(31.7)	94.3	<0.001
Type of comorbidity						
DM	862	215(24.9)	367	121(33)	8.50	0.003
HPT	935	202(21.6)	417	122(29.3)	9.37	0.002
Renal	370	96(25.9)	129	61(47.3)	20.28	<0.001
Heart	210	81(38.6)	65	40(61.5)	10.53	0.001
Lung	247	116(47)	88	60(68.2)	11.66	<0.001
Liver	61	33(54.1)	5	3(60)	0.07	0.80
Cancer	64	10(15.6)	11	3(27.3)	0.88	0.346
Other comorbidities	161	17(10.6)	168	62(36.9)	31.06	<0.001
At least one comorbidity	1596	341(21.4)	685	213(31.1)	24.49	<0.001
Number of comorbidities						
Nil	863	36(4.2)	419	69(16.5)	56.52	<0.001
One	706	94(13.3)	299	67(22.4)	12.92	<0.001
Two	544	111(20.4)	243	66(27.2)	4.45	0.035
Three	270	92(34.1)	113	53(46.9)	5.53	0.018
Four	76	44(57.9)	30	27(90)	9.93	0.001
DM + HTN	558	147(26.3)	225	75(33.3)	3.87	0.049
DM + HTN + Renal	112	47(42)	32	20(62.5)	4.18	0.004
DM + HTN + Heart	105	47(44.8)	31	17(54.8)	0.954	0.328
DM + HTN + Heart + Renal	17	8(47.1)	8	7(87.5)	3.55	0.059
Duration of hospital stays						
24 hours	21	7(33.3)	13	10(76.9)	5.93	0.015
24-72 hours	105	56(53.3)	80	40(50)	0.20	0.657
3-7 days	302	84(27.8)	230	72(31.3)	0.770	0.380
≥7 days	2031	230(11.3)	781	160(20.5)	39.99	<0.001

Distribution of COVID-19 risk score between first and second wave

The CSS score was calculated for the study patients (N=3563). Out of them, 65.9%, 26.9%, and 7.2% of patients were in the low (0-4), medium (5-7), and high (8-9) risk groups. In the first wave, a higher proportion of patients in the low-risk group (68.2% vs. 60.7%, p<0.001) and a lower proportion of patients in the middle-risk group (24.1% vs. 33.1%, p<0.001) were as compared to the second wave. In the higher-risk group (7.7% vs. 6.3%, p=0.137), an almost equal proportion of patients was in the first wave compared to the second wave.

**Figure 1:**
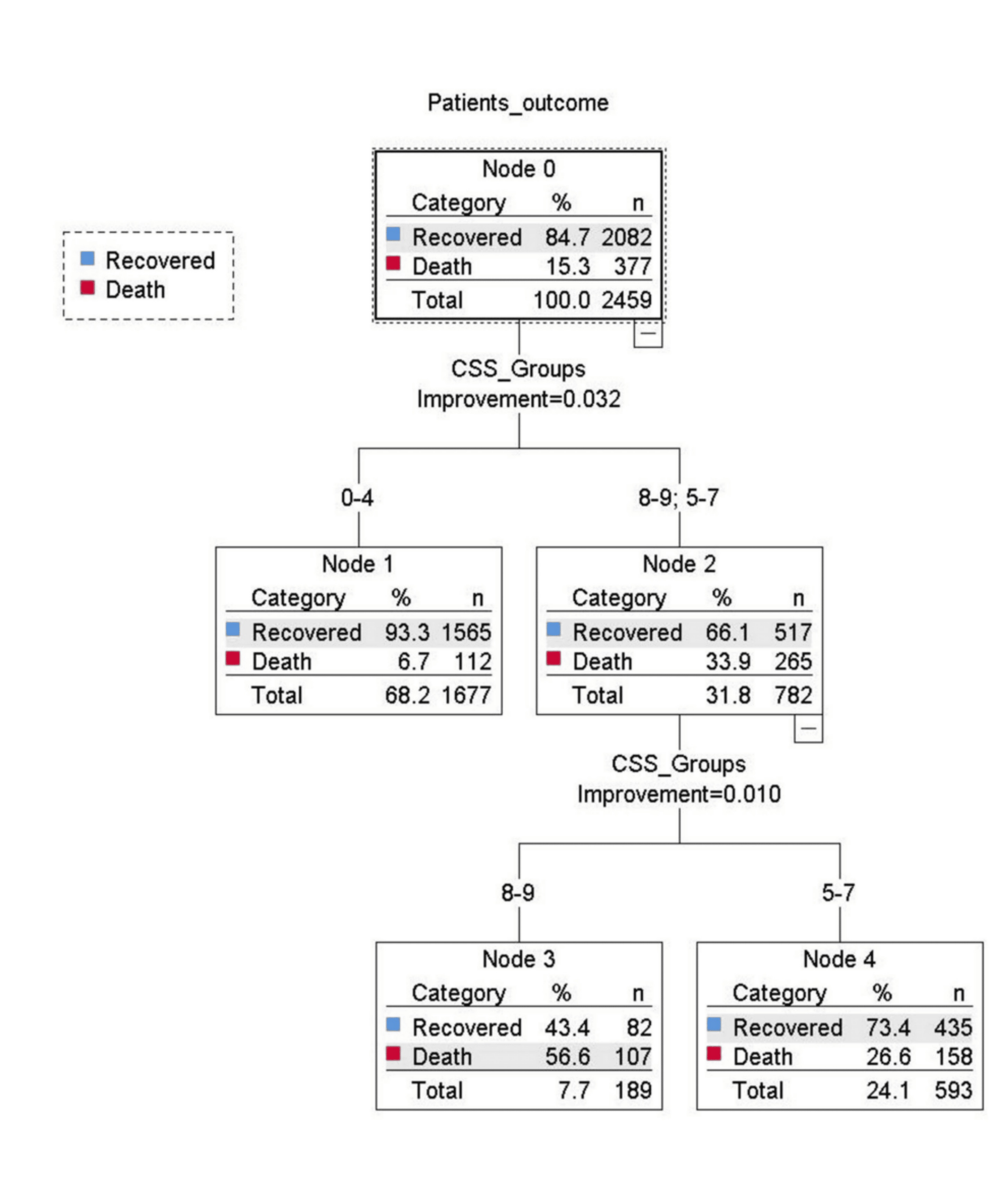
Classification and Regression Trees (CART) analysis showing the distribution of the patients mortality in three groups of the COVID-19 scoring system (CSS) during first wave of COVID-19.

**Figure 2:**
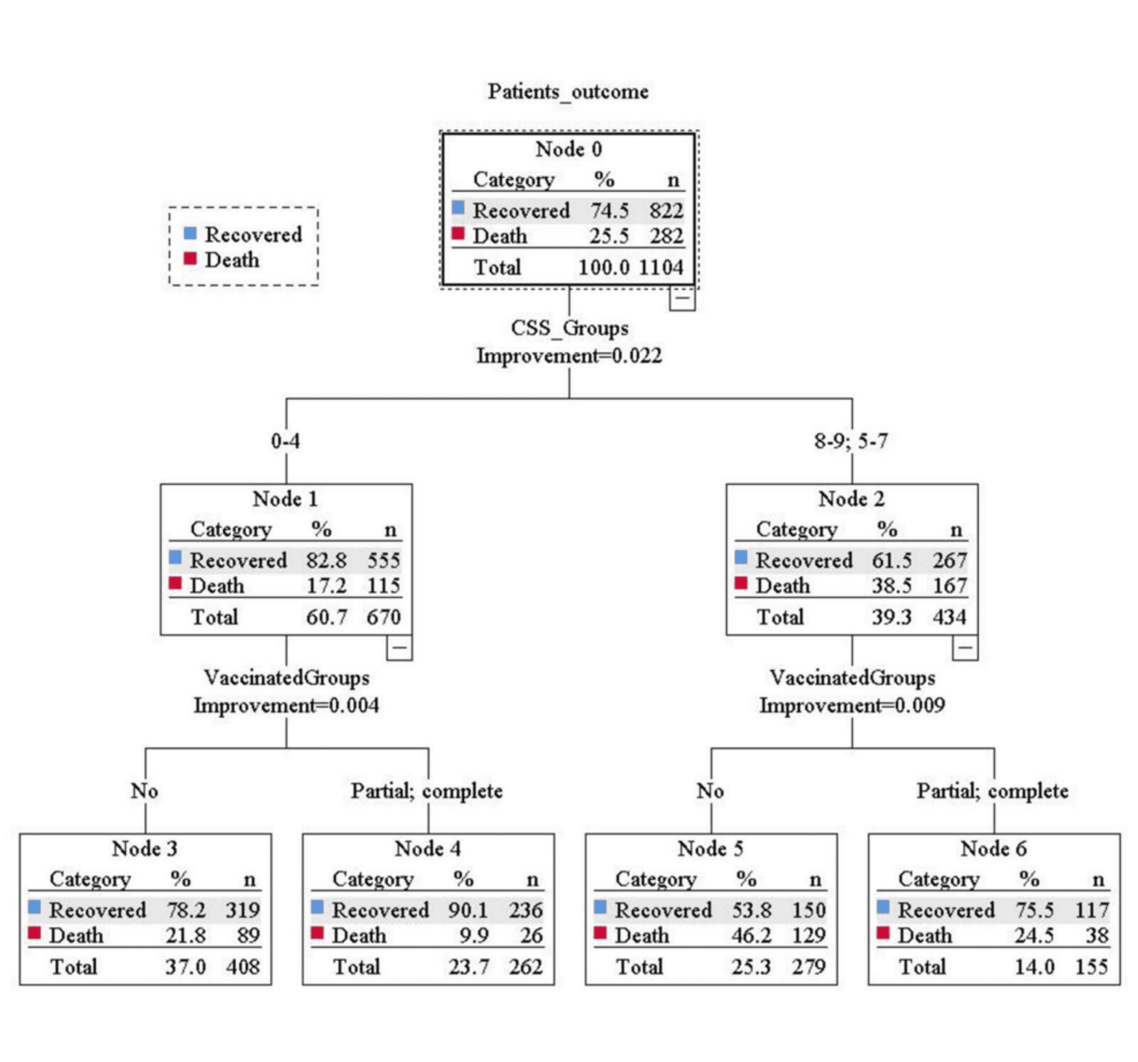
Classification and Regression Trees (CART) analysis showing the distribution of the patients mortality in three groups of the COVID-19 scoring system (CSS) during second wave of COVID-19 after including the effect of the vaccination.

**Figure 3:**
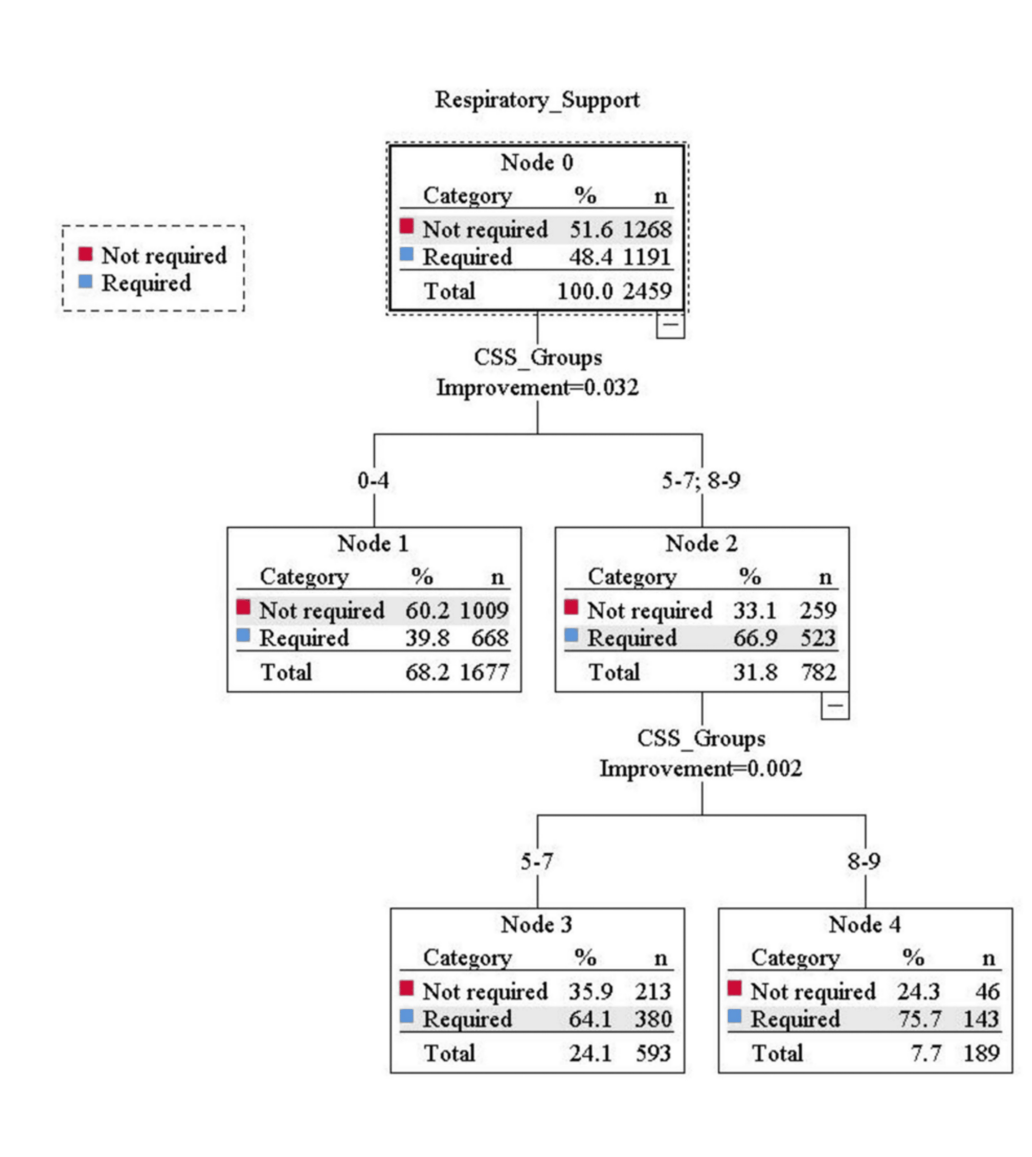
Classification and Regression Trees (CART) analysis showing the distribution of the required respiratory support in three groups of the COVID-19 scoring system (CSS) during first wave of COVID-19.

**Figure 4:**
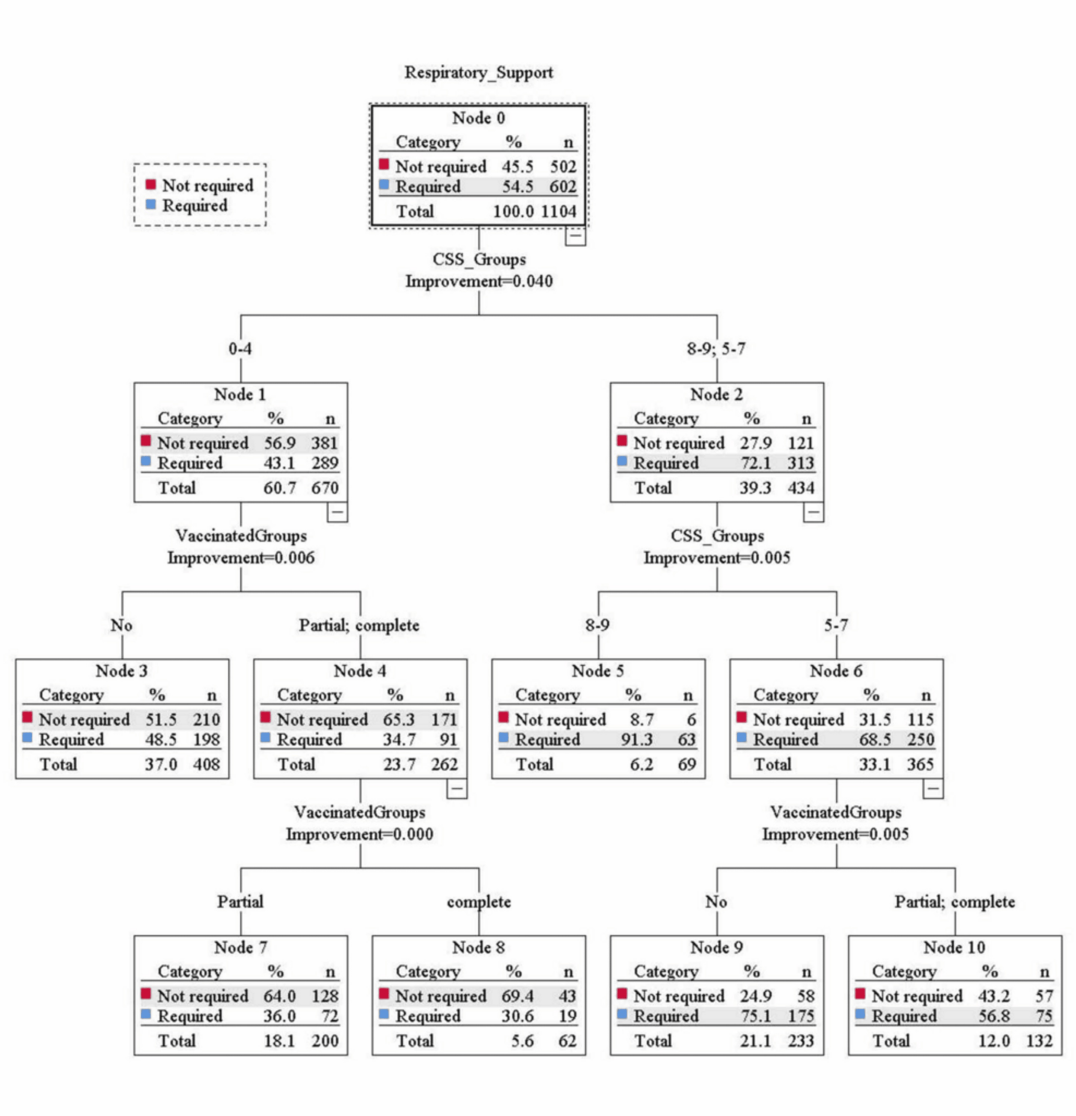
Classification and Regression Trees (CART) analysis showing the distribution of the required respiratory support in three groups of the COVID-19 scoring system (CSS) during second wave of COVID-19 after including the effect of the vaccination.

Binary logistic regression analysis evaluated the odds ratio for the risk of mortality and the need for respiratory support in three groups of CSS. The result showed that, during the first wave, there were 5.08 and 18.23 times the risk of mortality in the middle and high-risk groups, respectively, compared to the low-risk group. In the second wave, the risk of mortality was 2.58 and 6.81 times higher in the middle and high-risk groups, respectively, compared to the low-risk group after adjusting the effect of vaccination. Similarly for the need for respiratory support, during the first wave, there were 2.70 and 4.70 times the risk of required respiratory support in the middle and high-risk groups, respectively, compared to the low-risk group. In the second wave, the risk of required respiratory support was 2.86 and 13.92 times higher in the middle and high-risk groups, respectively, compared to the low-risk group after adjusting the effect of vaccination (Table 4 and Figures 1-4).

**Table 4:** Risk of mortality and need for respiratory support in middle and high-risk group patients in first and second waves (N=3563) L: lower limit, U: upper limit, univariable binary logistic analysis was used for first wave analysis, whereas ^#^multivariable binary logistic regression analysis was used for the second wave after including vaccination, which was not used in developing the COVID-19 scoring system (CSS). P<0.05 significant.

Variables	Odds ratio			Odds ratio		
	Value	95 % CI (L, U)	P-value	Value	95 % CI (L, U)	P-value
Outcome	Patient mortality			Respiratory support used		
First wave (n=2459)						
CSS groups			<0.001			<0.001
Middle-risk group	5.08	3.90, 6.61	<0.001	2.70	2.22, 3.27	<0.001
High-risk group	18.23	12.90, 25.76	<0.001	4.70	3.32, 6.64	<0.001
Low-risk group	Reference			Reference		
Second wave (n=1104) #						
CSS groups			<0.001			<0.001
Middle-risk group	2.58	1.91, 3.49	<0.001	2.86	2.18, 3.76	<0.001
High-risk group	6.81	4.00,11.60	<0.001	13.92	5.92, 32.76	<0.001
Low-risk group	Reference			Reference		
Vaccination			<0.001			<0.001
No vaccinated	3.12	1.60,8.09	0.001	2.61	1.61, 4.23	<0.001
Partial	1.25	0.61, 2.55	0.546	1.49	0.90, 2.48	0.124
Complete	Reference			Reference		

## Discussion

We reported and compared the demographic, epidemiological characteristics, and outcomes of hospitalized patients with COVID-19 between the first (n=2459) and second wave (n=1104), respectively. Patients in the second wave were older, with an increased proportion of females, clinical symptoms, and mortality as compared to the first wave. Almost equal proportions of comorbidity were observed between the two waves. Double-dose vaccination was effective in reducing the mortality and requirement for respiratory support and was more effective than a single dose. In the first wave, a significantly higher proportion of patients were in the low-risk group and a lower proportion of patients in the middle-risk group, whereas an insignificantly higher proportion of patients were in the high-risk group compared to the second wave. The literature comparing and focusing on differences between India's first and second waves is limited. The basic characteristics of the first and second waves of COVID-19 have shown that infectivity, oxygen and ventilator support, positivity rate, disease spread rate, involvement of younger patients, and death rate were higher in the second wave [9]. The severe clinical symptoms occurred in a greater proportion of cases during the first wave as compared with the second wave (27.8% vs. 10.6%, P = 0.03), although the author did not explore the mortality and other outcomes in the patients [10]. Iftimie et al. analysed 204 and 264 patients hospitalized during the first and second waves, respectively. The results showed that in the second wave, patients were younger (also affected more children) and had a lower duration of hospitalization and case fatality rate. The presence of comorbidities like cardiovascular diseases, type 2 diabetes mellitus, and chronic neurological diseases was similar in both waves. Patients from the second wave more frequently had renal and gastrointestinal symptoms and had a higher requirement for non-invasive ventilation [11]. Emerging preprint data from a study comparing the COVID-19 mortality rate in the first and second waves from India has shown a greater than 40% increase in mortality and noted an alarming finding of the highest rate of mortality among people younger than 45 years of age [12]. Data from 14 high-income countries showed that there was a small absolute difference in COVID-19 deaths of individuals aged <50 years between the two waves (absolute difference range: 0-0.4%). The proportion of youth dying in the second wave was lower than in the first wave (prevalence ratio 0.81, 95% CI 0.71-0.92), although there was considerable heterogeneity between countries. Similarly, the proportion of deaths <70 years of age was lower in the second wave (prevalence ratio 0.96, 95% CI 0.86-1.06), although the result was statistically insignificant [13]. In the second wave, the Delta variant (B.1.617.2 strain) was more highly contagious and rapidly spreading than the Alpha variant in the first wave. The Delta variant was 50% more contagious and had an increased risk of hospitalization than the Alpha strain. There are more than 12,200 variants of concern in the country, as shown by genomic sequencing, but their presence is minuscule compared to the Delta variant, which replaced all other variants in the second wave [14-17]. Compared to the first wave, the second wave of the pandemic reported a higher proportion of deaths, with COVID-19 pneumonia and respiratory failure emerging as major causes of death. In contrast to the first wave, the second wave of the pandemic observed fast disease progression and pneumonia, including among the young [18-20]. Most of the comparisons conducted between the first and second waves showed that there was a higher proportion of younger patients, clinical symptoms, respiratory support, and mortality in the second wave. In our study, except for age, which was higher in the second wave, the rest of the findings were similar to other studies [9-13]. In our study, the reason for the older population in the second wave can be attributed to the sudden increase in the number of patients during the short period of April to May 2021. Our tertiary care referral centre usually admitted patients who were at high risk, older age groups, whereas, in the first wave, patients were admitted mainly from May 2020 to January 2021, and due to larger intervals in duration, all age group patients had a chance of admission.

In India, overall COVID-19 mortality is around 1.3%, whereas in our centre it was 15.3% and 25.5% in the first and second waves, respectively. The higher mortality reflects patients with a more severe disease, with approximately half of them requiring any respiratory support and having co-morbidities.

Strength and limitation of the study

The present study was conducted on a very large number of 3563 patients, and the evidence generated from the study may be useful to understand the severity of the new variants, and it would guide policymakers to be alert to the new variants and take necessary preparations as a risk mitigation process. There were significant differences in demographics, epidemiological risk factors, disease severity, respiratory support, and outcomes between the two waves, indicating an impact of the new variants on existing variants. The limitation of the study was that most of the patients admitted to our dedicated COVID-19 tertiary care facility were referred from various district hospitals or state COVID-19 care help desk centres; hence, they required more respiratory support than usual and had a higher mortality rate.

## Conclusions

Our study highlights some important differences as well as similarities between the first and second waves of COVID-19 in Indian patients. The study has certain key limitations. Laboratory data, treatment strategies, and follow-up of patients were not analysed. Furthermore, by virtue of being a referral centre, the majority of the patients had moderate-to-severe disease, thus possibly not representing the entire spectrum of COVID-19.
